# The Duality of Dioxolanyl Radicals towards C–C Bond Construction

**DOI:** 10.1055/a-2599-8435

**Published:** 2025-05-19

**Authors:** Kyra L. Samony, Justin J. Chang, Daniel K. Kim

**Affiliations:** Temple University, Department of Chemistry, 1901 North 13th Street, Philadelphia, Pennsylvania 19122, USA

**Keywords:** acetal, dioxolane, radical, HAT, decarboxylation

## Abstract

Protecting groups are commonplace in organic synthesis and appear often in the form of dioxanes and dioxolanes. 1,3-Dioxolanes can be used for the protection of both diol and carbonyl motifs. Our group has been increasingly interested in alternative uses for such a simple and often overlooked heterocycle. Herein we highlight recent advancements in dioxolanyl radical chemistry where reactivity can be selectively directed towards two unique positions leading to distinct product formations, resulting in trifluoromethyl ketones and 1,2-diol functionalities.

## Graphical Abstract

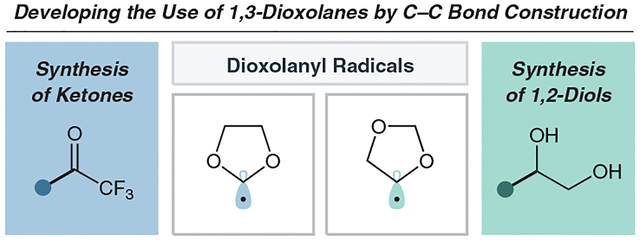

## 1 Introduction

**Figure 1:**
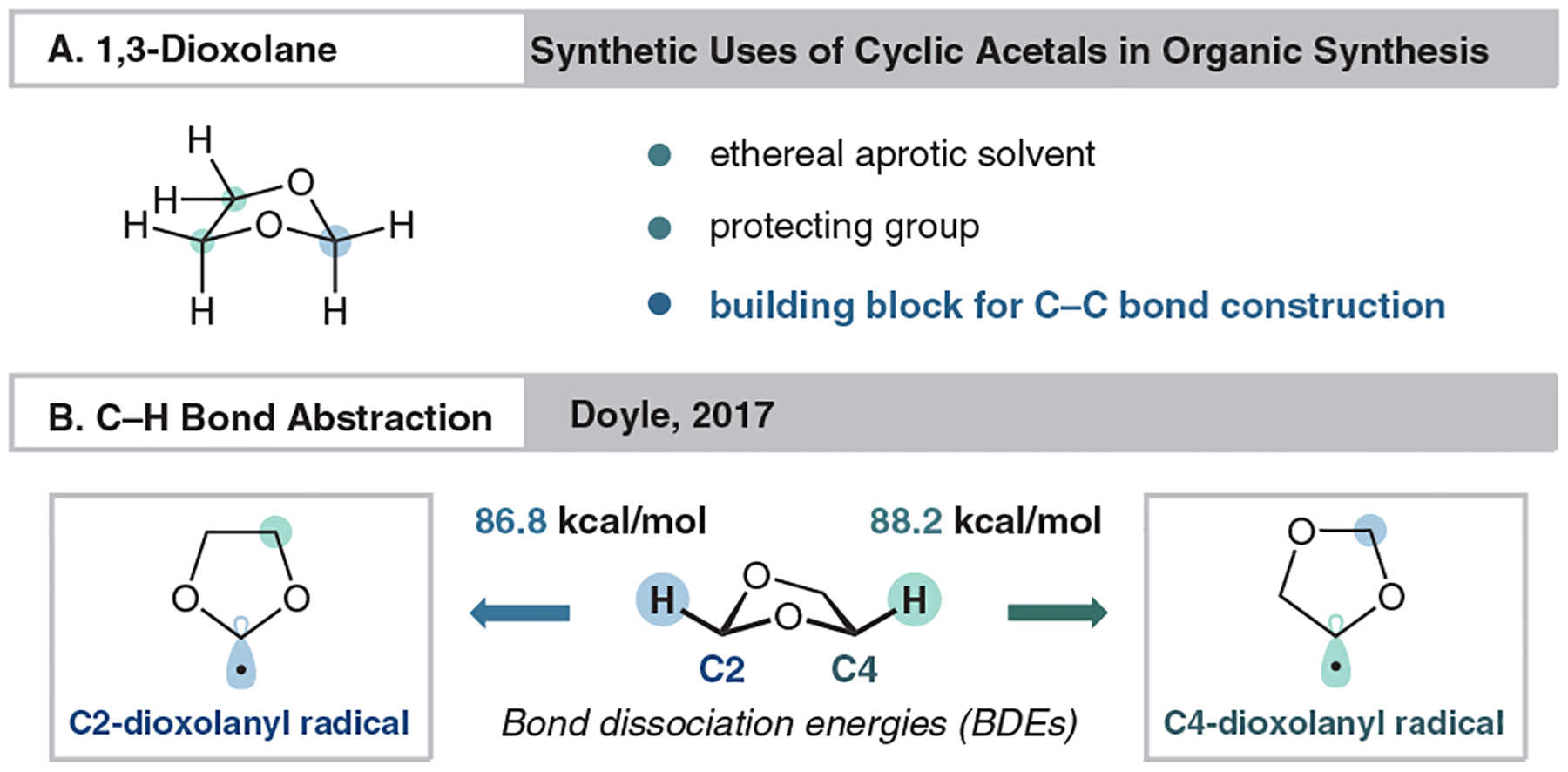
Dioxolane general uses and bond-dissociation energies (BDEs)

Dioxolanes appear often within the context of both classical and modern organic chemistry in largely one of two simple ways, as either a reaction solvent or protecting group. While commonplace in both synthesis and methodology, dioxolanes are often overlooked in conversations centered around the discovery and development of advanced synthetic strategies. Such a simple structure can be activated to yield a multifunctional carbon-bond framework, poised for the direct introduction of oxygen-containing functional groups such as aldehydes, ketones, and free diols, while circumventing traditional oxidation chemistry (Figure 1A).

Historically, fully saturated cyclic ethers, such as furans, dioxolanes, pyrans, and dioxanes, have been employed as aprotic solvents for organic reactions from as early as the late 19th century.^1^ These ethers are known for their low boiling points, low reactivity, and relative polarity, and are an important class of nonaromatic heterocycles. Dioxolane, specifically 1,3-dioxolane, has been and continues to be employed as an environmentally friendly and cost-conscious substitute to other ethereal solvents like THF or diethyl ether.^2,3^

Aside from its use as reaction media, dioxolane is most famously known to be employed in protection strategies for labile oxygen-containing functional groups.^4–6^ Diols, ketones, and aldehydes can be easily masked by inexpensive and commercial reagents such as acetone and ethylene glycol, respectively, and are just as easily deprotected in mild acidic conditions.^7,8^ The capability of such a functional group to protect both carbonyls and glycols in equal ease highlights its duality and wide applicability. Facile syntheses coupled with low cost and minimal hazard make dioxolanes an effective and obvious choice for shielding reactive species. Dioxolanes as protecting groups are long-established in literature, ranging from protections of complex carbohydrates to key fragments in natural product syntheses.^9–12^

Building upon their role in traditional protection strategies, it has been documented that dioxolanes also have success in radical-mediated reactions, enabling the synthesis of high-value masked carbonyls. These desired radicals can be generated through a variety of means, such as hydrogen atom transfer (HAT) or oxidative decarboxylation processes, which we discuss extensively in our review of acetal radicals and their applications.^13^

In a study by Doyle and coworkers, the abstraction of C–H bonds on solvent-grade 1,3-dioxolane was explored (Figure 1B).^14^ Intriguingly, radical formation at both the 2- and 4-positions was observed. The C–H bond at the 2-position has a lower bond-dissociation energy (BDE) of 86.8 kcal/mol, compared to the 4-position of 88.2 kcal/mol, and the authors note that there is a preference to the more accessible BDE. This leads to the observed selectivity for radical formation at one position over the other, mediated by a photoredox-generated HAT reagent. This report provides a foothold to demonstrate potential functionalization at each respective position.

The overall need for protection strategies stems from the fundamental issue of chemoselectivity as molecules become more complex.^15^ An approach that leverages both the intrinsic reactivity and polarity of diols and carbonyls would be extremely valuable to the synthetic community. With this consideration in mind, we postulate that 1,3-dioxolane motifs can act as highly oxidized carbon constituents ideal for fragment coupling. The duality of dioxolanyl radicals lends itself well to establishing a general reagent that can access both diols and carbonyls through controlled chemoselective radical generation at either of the aforementioned positions. We proposed employing these dioxolane skeletons as specially tuned synthetic handles towards radical C–C bond construction. This method is uniquely situated to aid in the installation of highly oxidized functional groups such as ketones, aldehydes, and alcohols. Just as dioxolanes can be employed in dual protecting strategies, dioxolanyl radicals can be thought of as dual-use coupling fragments that will facilitate the construction of highly polar complex molecular scaffolds.

With this design strategy in hand, our lab has recently designed a fluorinated dioxolane-based carboxylic acid that enabled an otherwise challenging coupling reaction, in this case, at the dioxolane 2-position, accessed by oxidative decarboxylation, to construct highly sought after trifluoromethyl ketones (TFMKs).^16^ Following the success of our initial findings, we continued our exploration of dioxolanyl radicals instead at the 4-positon to install highly polarized and valuable vicinal diols through a direct photomediated hydrogen atom transfer (HAT) process.^17^

## 2 Masked Trifluoromethylacetylation

A cornerstone of our research program relies heavily on dioxolanyl-radical based strategies to form C–C bonds. We had broadly aimed to develop a new methodology to incorporate TFMKs into complex molecular scaffolds. Fluorine-containing coupling fragments are becoming an essential component in small-molecule drug discovery, with fluorine appearing in many of the best-selling agrochemicals and FDA-approved therapeutics.^18–20^ As such, the synthetic community would greatly benefit from an arsenal of complementary synthetic strategies to access a variety of fluorofunctional groups.

In particular, TFMKs are of interest due to their ability to enhance desired pharmacokinetic properties such as increased lipophilicity, metabolic stability, and enzyme inhibition.^21–25^ It is well-reported that TFMKs, in both their ketone and hydrate form, act as bioisosteres to carboxylic acids, which again can help alleviate undesired biological responses.^26,27^ Many traditional approaches to synthesizing TFMKs rely on two-electron chemistry, adding various nucleophiles into fluorinated electrophiles such as trifluoroacetic anhydride, ethyl trifluoroacetate, or fluorinated Weinreb amides.^28^ We noticed that there was a need for an alternate disconnection and began developing a nucleophilic TFMK reagent which would allow us to install TFMKs into medicinally relevant electrophilic substrates such as strained bicycles, imines, and heteroaromatics. While we were exploring this, a radical-based photochemical approach was disclosed by Katayev and coworkers wherein trifluoroacetic anhydride was reduced to yield a trifluoromethyl acyl radical by C–O fragmentation to put forth in coupling to electron-rich aryl olefins.^29^ We were able to expand upon their strategy with our own, in turn furnishing access to electron-deficient partners, thus yielding a complementary radical approach towards TFMK synthesis.^16^

**Figure 2:**
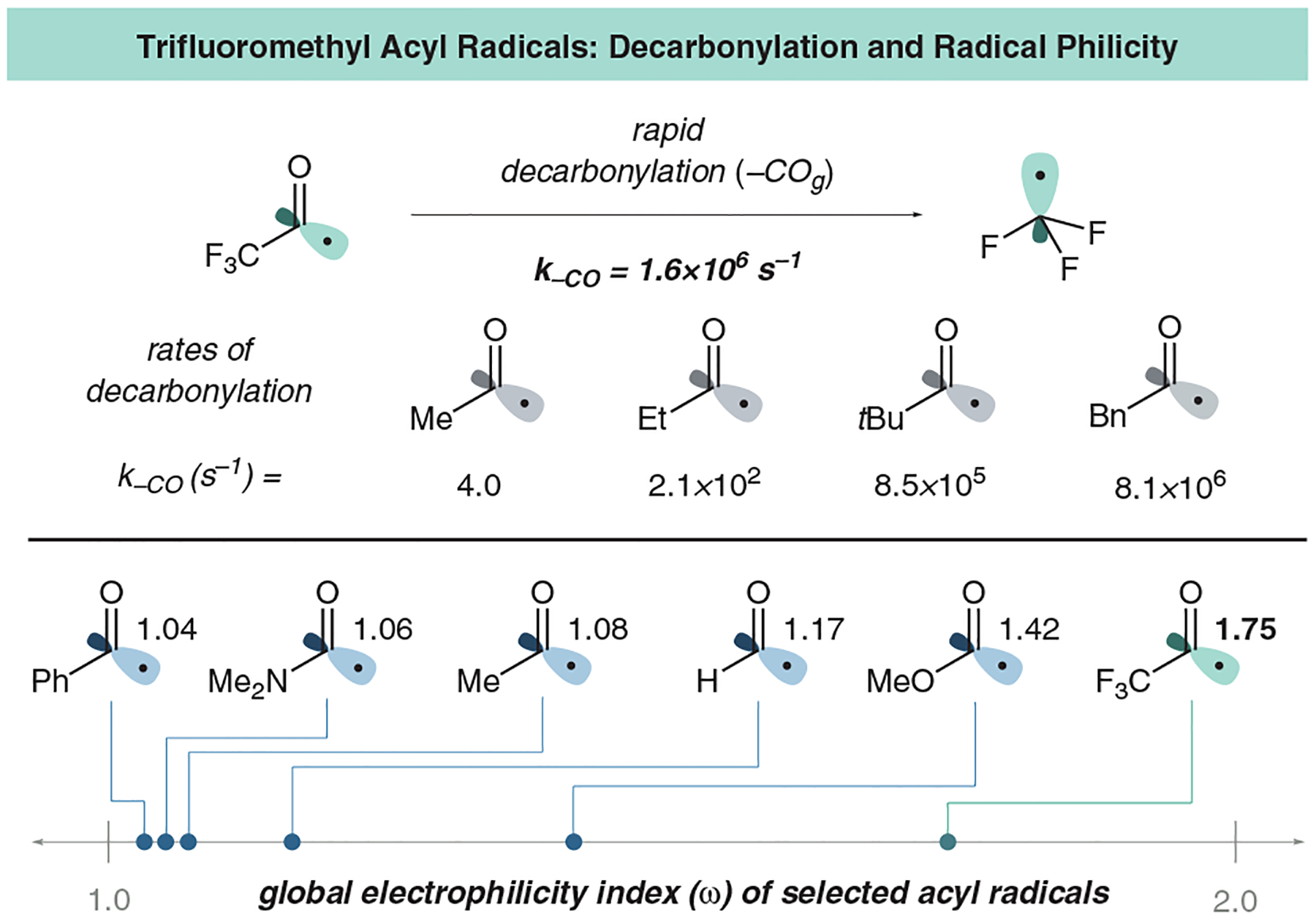
Decarbonylation rates and electrophilicity values for common acyl radicals

Trifluoromethyl acyl radicals are prone to rapid decarbonylation and are thus difficult to harness for coupling.^30^ They are also exceptionally electrophilic when compared to other acyl radicals, with a global electrophilicity value calculated to be 1.75 by Katayev and coworkers (Figure 2).^31^ This is considerably more electrophilic than that of the methyl acyl radical with a value of 1.08, calculated by Nagib and coworkers.^32^ Considering the relative electrophilicity of trifluoromethyl acyl radicals, reactivity towards electron-deficient partners would be challenging, even without the added issue of decarbonylation. This premise is further supported experimentally by our inability to couple trifluoropyruvic acid to electron-deficient olefins.^16^

**Figure 3:**
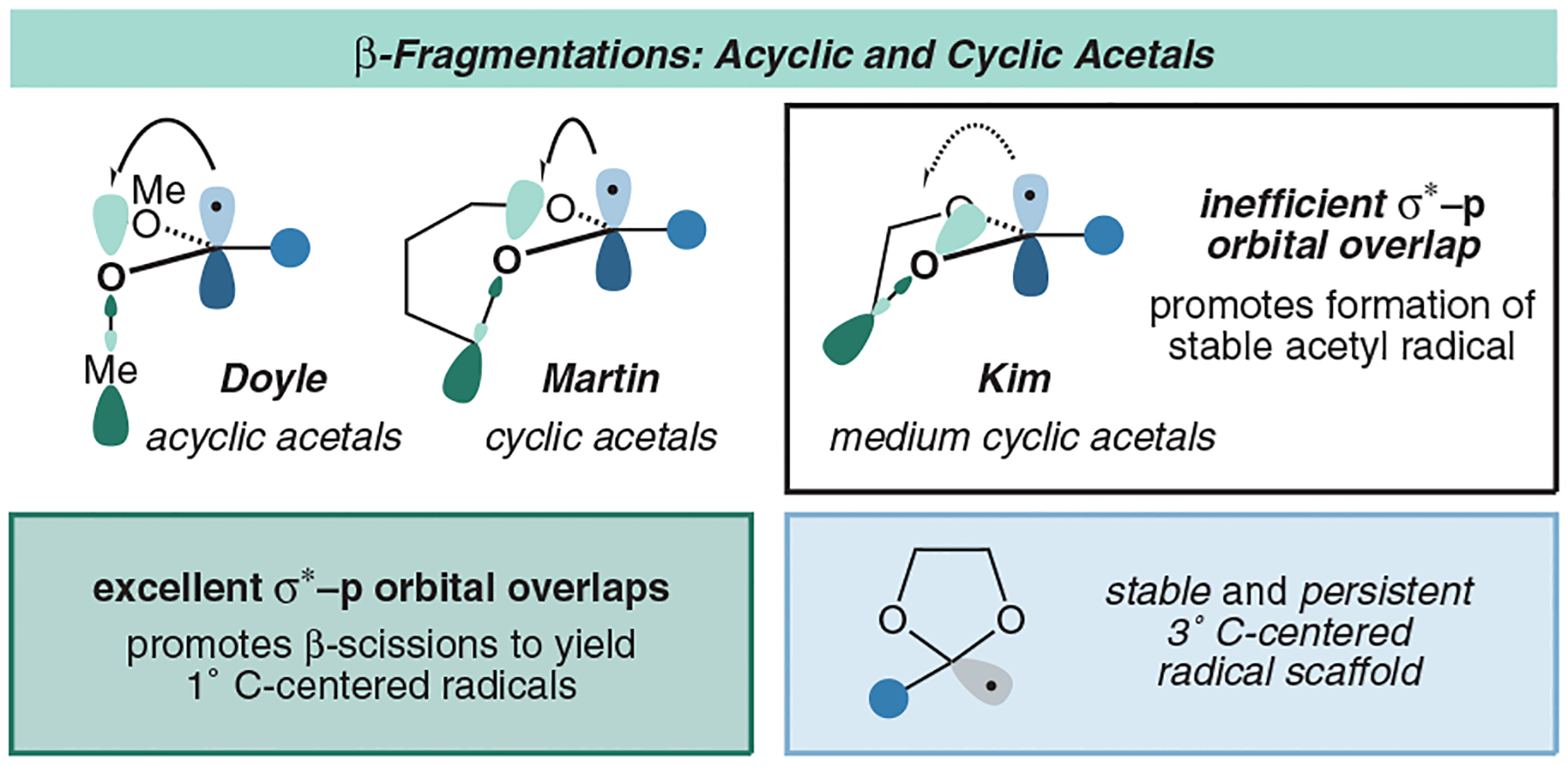
Circumventing β-fragmentation pathways in reagent design

With all of this in mind, we concluded that we could solve both issues, radical polarity and stability, by installing some type of masking group. For simplicity, we envisioned putting forth conventional carbonyl-protection strategies that would promote nucleophilic radical generation, such as the introduction of a stabilizing ether. It is well-precedented that C-centered radicals can be formed at the α,α - dialkoxyalkyl position of both cyclic and acyclic acetals.^13^ It was imperative to design an ethereal skeleton that would yield a radical that was stable and persistent enough for coupling without further rearrangement, as many of these radicals are known to promote β-fragmentation pathways.^33–37^ Doyle and coworkers have done extensive studies on the generation of methyl radicals from acyclic trimethyl orthoformate, which is promoted through excellent σ*–p orbital overlap.^38^ In addition to acyclic systems, Martin and coworkers disclosed an informative report that highlights the orbital overlap and likelihood of β-scission in cyclic dioxolanes, dioxanes, dioxepanes, and dioxocanes.^39^ It was found that the conformational flexibility afforded to larger seven- and eight-member cyclic acetals enabled the σ*–p orbital overlap to promote β-cleavage (Figure 3), while the more inflexible five- and six-member rings yielded only the tertiary acetal radical.

**Figure 4:**
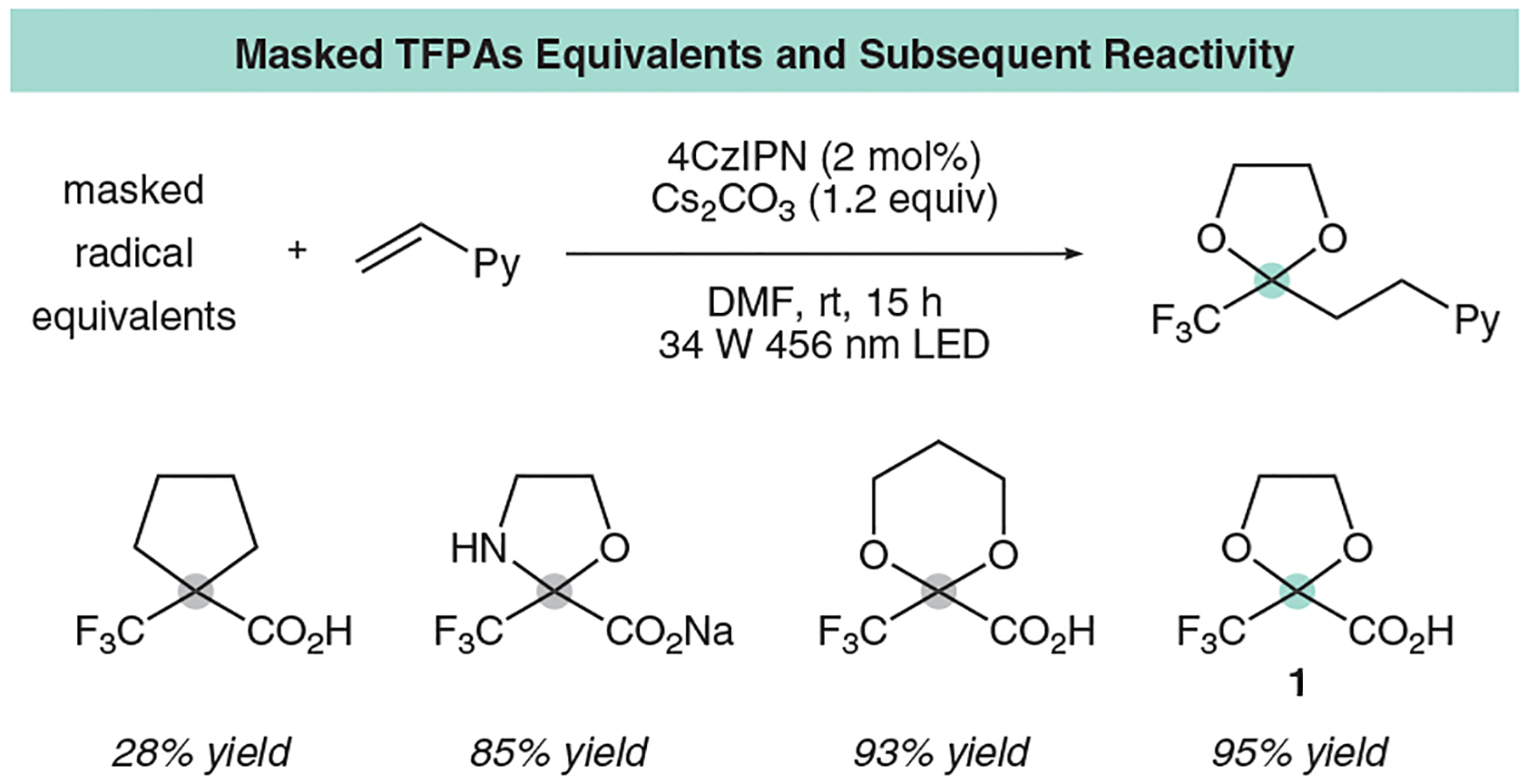
Initial design strategies and designer radical equivalent reagents

With the ability to circumvent β-fragmentations, as well as enhanced stability and in turn nucleophilicity, the five-membered dioxolane was targeted as an ideal scaffold and thus inspired a variety of fluorinated carboxylic acids (Figure 4), with varying alkyl and heterocyclic substituents, such as cyclobutane, oxazolidine, dioxane, and dioxolane, which were all easily synthesized in two steps.

**Figure 5:**
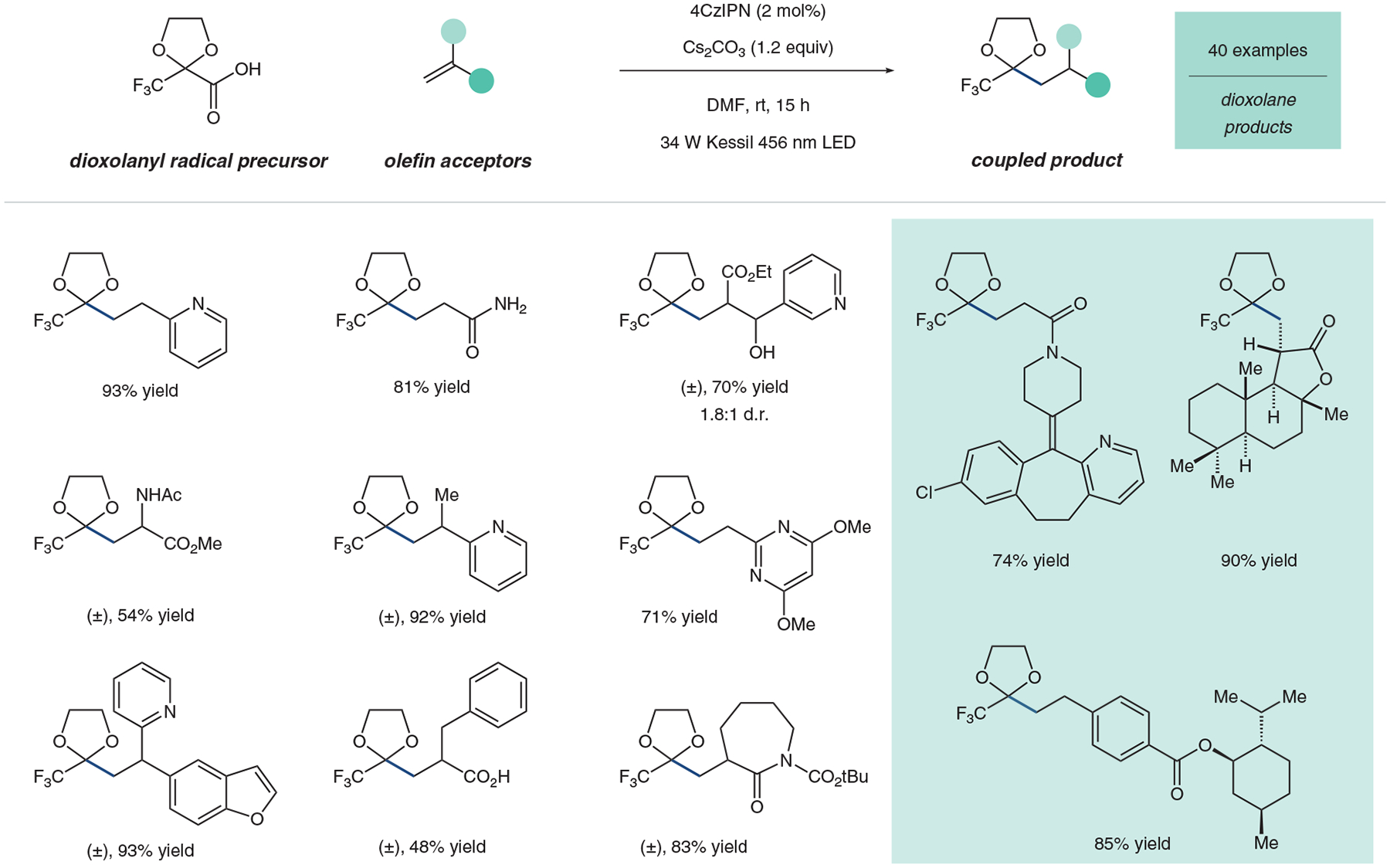
Highlighted substrate scope for the incorporation of the trifluoromethyl dioxolanyl radical reagent

Interestingly, the dioxolanyl-based structure **1** proved best for coupling with 95% coupling yield with model substrate 2-vinylpyridine through a traditional oxidative decarboxylation followed by addition into the olefin and subsequent reduction and protonation. Following our initial report, the Katayev group has also calculated the electrophilicity value of the resulting trifluoromethyl dioxolanyl radical to be 1.01, again reinforcing the improved nucleophilicity of our trifluoromethyl dioxolanyl radical.^31^ We used this reagent to couple to forty electron-deficient alkenes and vinyl heterocycles, with a selected scope highlighted here to showcase the reactivity of our dioxolanyl radical (Figure 5). Excitingly, these mild conditions tolerated complex substrates like loratadine, menthol, and sclareolide.

**Figure 6:**
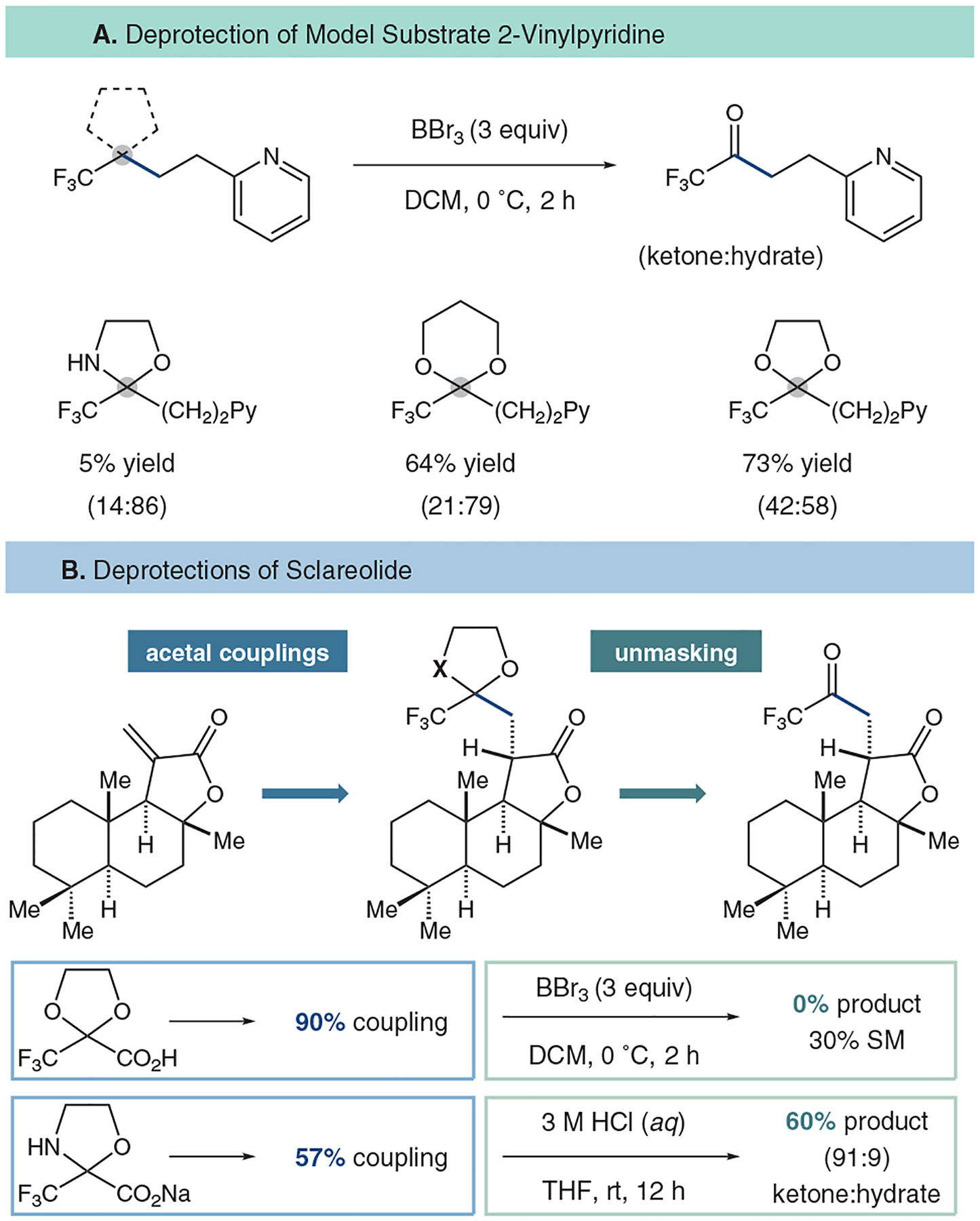
Deprotection strategies towards accessing TFMKs

Subsequently, the dioxolanyl-containing compounds could be deprotected to yield the highly sought-after TFMKs using Lewis acidic boron tribromide which was demonstrated on our model substrate (Figure 6A). The unmasking was uniquely challenging and did not proceed using traditional dioxolane-removal strategies. For sensitive substrates, like sclareolide, alternative masking agents can be engaged, although with loss of yield in the coupling (Figure 6B).

The data collected in our initial study supports that electron-donating atoms enable more nucleophilic character of the resulting dioxolanyl radical, creating the desired electron-rich ‘acyl’ radical. An interesting result from this study shows that the oxazolidine-based reagent had decreased yields when coupling to olefins and other electrophilic partners, while presumably possessing more electron donation from the nitrogen atom. We are currently investigating the unique properties and reactivity of the generated radical through further experimentation and computations.

The use of our trifluoromethyl dioxolanyl radical promoted chemical reactivity that has been otherwise challenging through other more traditional hydroacylation strategies. The twofold use of dioxolanyl radicals as both a stabilizer and inverse polarity facilitator was essential to the success of this study. Since our seminal report, other groups have employed our masked radical equivalent towards couplings to specialized heterocycles and alkenes.^40,41^ While this reinforces that we have developed a synthetically useful reagent which promotes a highly desired transformation to new electrophiles, the deprotection still necessitates the use of boron tribromide to access the resulting TFMK. In many contexts, the use of boron tribromide as an unmasking reagent is a reasonable way to reveal the desired TFMKs. However, in the case of Lewis acid sensitive functional groups, or necessity on industrial scale, its use as an unmasking reagent can be too harsh. Since our initial finding, we have been working towards milder and more general deprotection strategies to access TFMKs from other electrophiles. Additionally, specifically regarding Minisci-type reactivity, we successfully developed a second-generation masked radical equivalent which lends itself to deprotection much more easily and without the need for strong Lewis acids or bases.

## 3 Dioxolanyl Radicals: Vicinal Functionality

**Figure 7:**
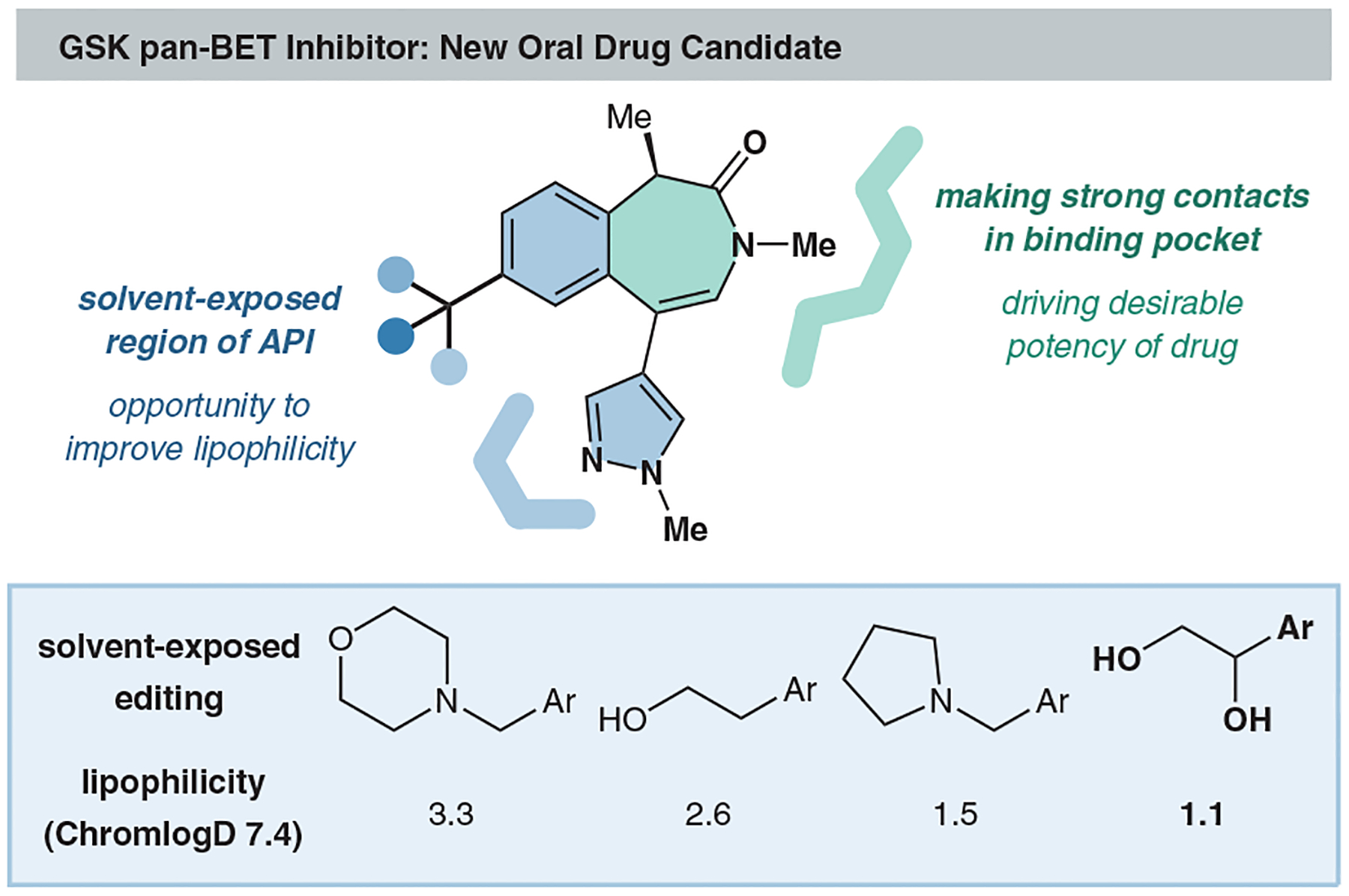
Vicinal diols in drug synthesis

Our group successfully employed a dioxolane-based designer reagent as both a masking agent and inverse polarity inducer for the introduction of the trifluoromethyl ketone moiety.^16^ While concluding our initial study, we considered other possible uses for the dioxolane core. We saw an opportunity for dioxolanyl radicals to be used to install another highly oxidized functional group, 1,2-diols, which are often protected into dioxolanes in traditional syntheses. Highly polarized fragments like vicinal diols can introduce favorable hydrogen-bonding interactions in enzymatic binding pockets and improve aqueous solubility, which in turn improves their overall pharmacokinetic profile.^42,43^ Additionally, internal hydrogen bonding helps to preserve drug permeability, which can rapidly deteriorate in the presence of other highly polar functional groups.^43^ When targeting pan-BET bromodomain inhibitors, GSK scientists found that the introduction of a diol motif in lieu of the traditional alcohol helped improve aqueous solubility while maintaining suitable membrane permeability (Figure 7).^44^ As such, pharmaceutical companies like GSK, Novartis, Genentech, and Argenta have used this to great effect in various enzyme inhibitors.^44–46^ Additionally, 1,2-diols are synthetically useful intermediates to rapidly build molecular complexity from simple building blocks.^47^

**Figure 8:**
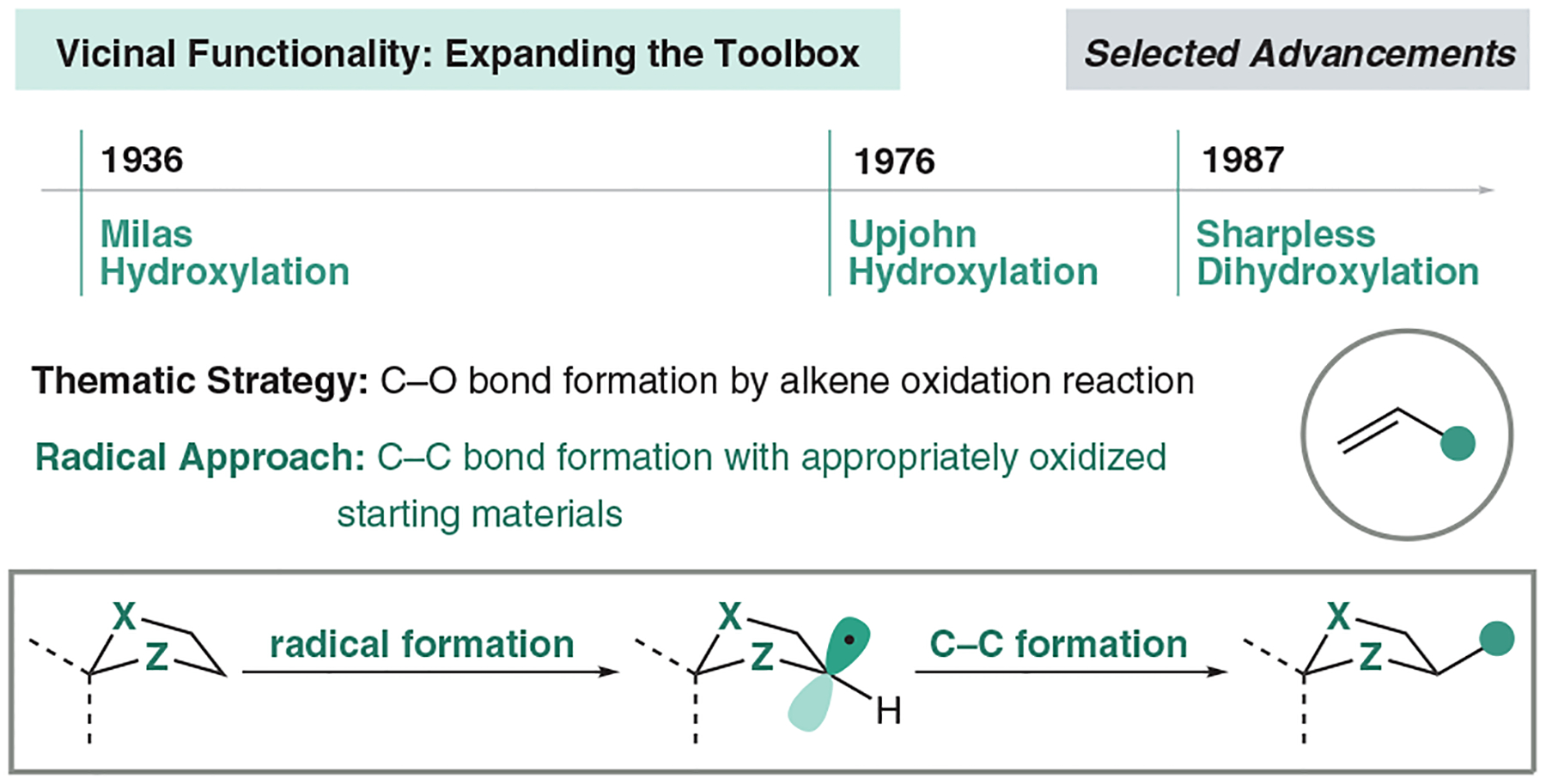
Advancements in vicinal diol synthesis

In 1936, Nicholas Milas and Sidney Sussman reported the use of osmium tetroxide as a means to oxidize alkenes to *syn*-diols.^48^ Nearly a century later, olefins are still highly used to facilitate the desired dihydroxylations. Since this seminal report, many synthetic groups have optimized and refined this oxidation for milder reaction conditions and stereoselective control.^49^ These efforts culminated in the Nobel Prize winning Sharpless dihydroxylation, demonstrating how alkenes have become the premier precursor to access this substructure in both natural product total synthesis and small-molecule drug design.^47,49^ Further expanding upon this strategy, synthetic chemists have used alkenes as a precursor to other vicinally functionalized motifs like 1,2-diamines, 1,2-amino alcohols, and 1,2-thiol alcohols through oxidative pathways.^50–52^ In the early stages of development in our dioxolanyl radical chemistry, we saw an opportunity to install 1,2-diols in a complementary C–C bond-forming approach via hydrogen atom transfer (HAT) catalysis (Figure 8).^17^

**Figure 9:**
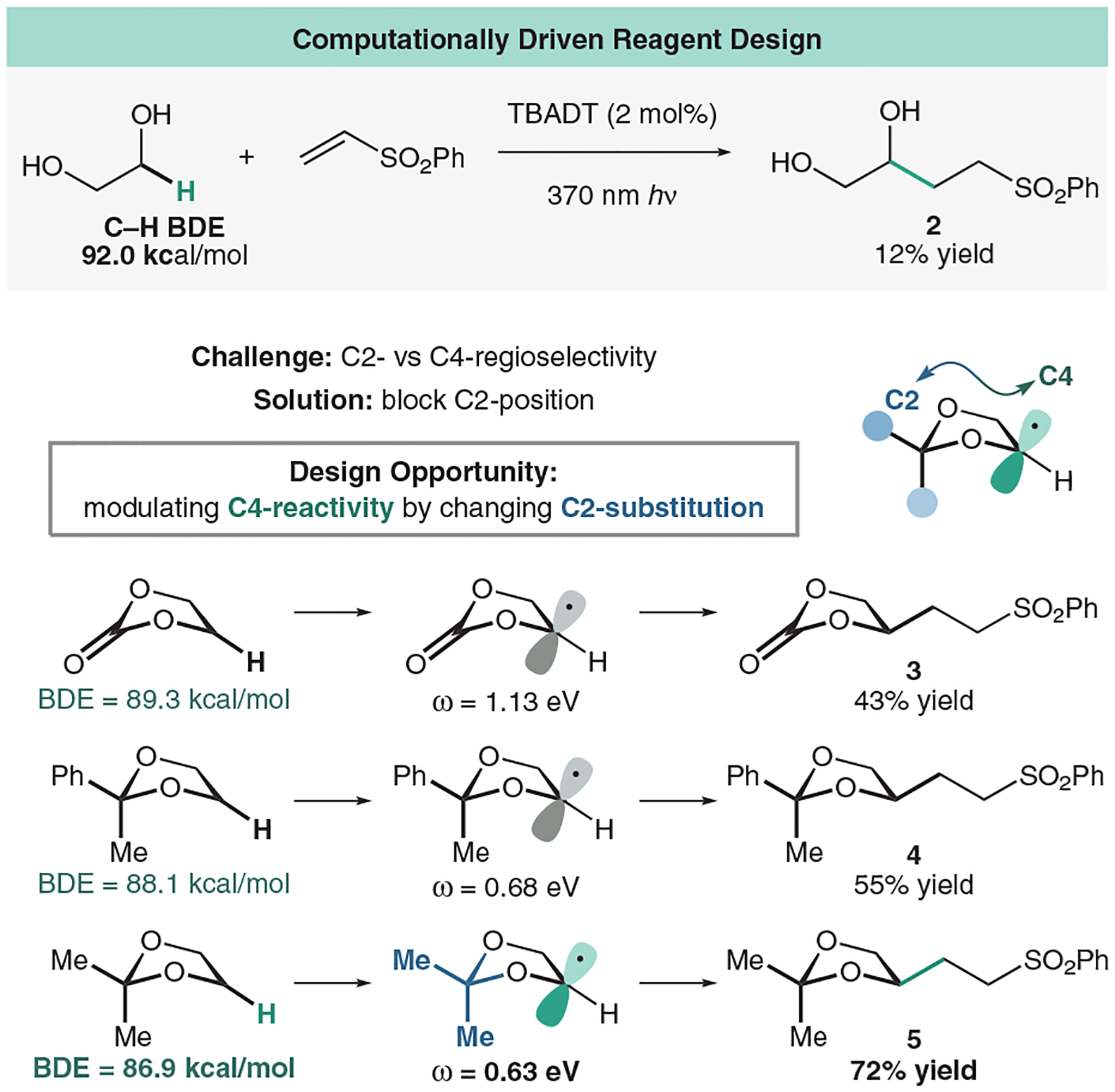
Using computations to design reagents

In our initial study, we attempted to use ethylene glycol directly as a cheap and readily available reagent for the desired C–C bond construction with model olefin phenyl vinyl sulfone. Interestingly, this reaction proved to be very inefficient with only 12% conversion to product after 16 h of irradiation with 370 nm light (**2**). We envisioned that, similar to our previous study involving the trifluoromethyl dioxolane reagent, that a dioxolane scaffold could be used as an activator of ethylene glycol to bolster this reactivity (Figure 9).^53^ We hypothesized that we could use C–H BDEs as a way to quickly evaluate masked diol reagents. When calculating the C–H bonds of 1,3-dioxolane, we found that the 2-position was the weakest (86.8 kcal/mol), followed by the 4-position (88.2 kcal/mol), which is also in accordance with the report from Doyle and coworkers.^14^

These slightly differing values can be due to a hyperconjugation effect from the flanking oxygens at the 2-position, which in turn help in stabilizing the radical intermediate. Additionally, Doyle and coworkers confirmed that C–H abstraction does occur at the 2-position of the dioxolane over the 4-position at about a 9:1 ratio, a selectivity in line with the difference in BDE between the two positions (1.4 kcal/mol).^14^ Because of this insight, we designed our acetal reagent to block the 2-position of the dioxolane for sole regioselectivity at the 4-position. Excitingly, moving to simple ethylene carbonate improved our yield to 43% (**3**). We recognized that we could use thermodynamic BDEs as a measure to roughly estimate reactivity. However, it was not obvious how distal changes at the 4-position might impact the BDE at the 2-position. Thus, we turned to computational analysis for a systematic approach to reagent design. We explored many possible contenders, computing the BDE and subsequently exploring the coupling experimentally (**3**). Simple screenings of acetal reagents by swapping ketones in an acetal condensation yielded great differences in C–H BDE and subsequently coupling yields (**4**). We found that simple 2,2-dimethyl-1,3-dioxolane, derived from readily available, green, and affordable ethylene glycol and acetone, was the best reagent for our desired transformation, affording a 72% yield (**5**).

**Figure 10:**
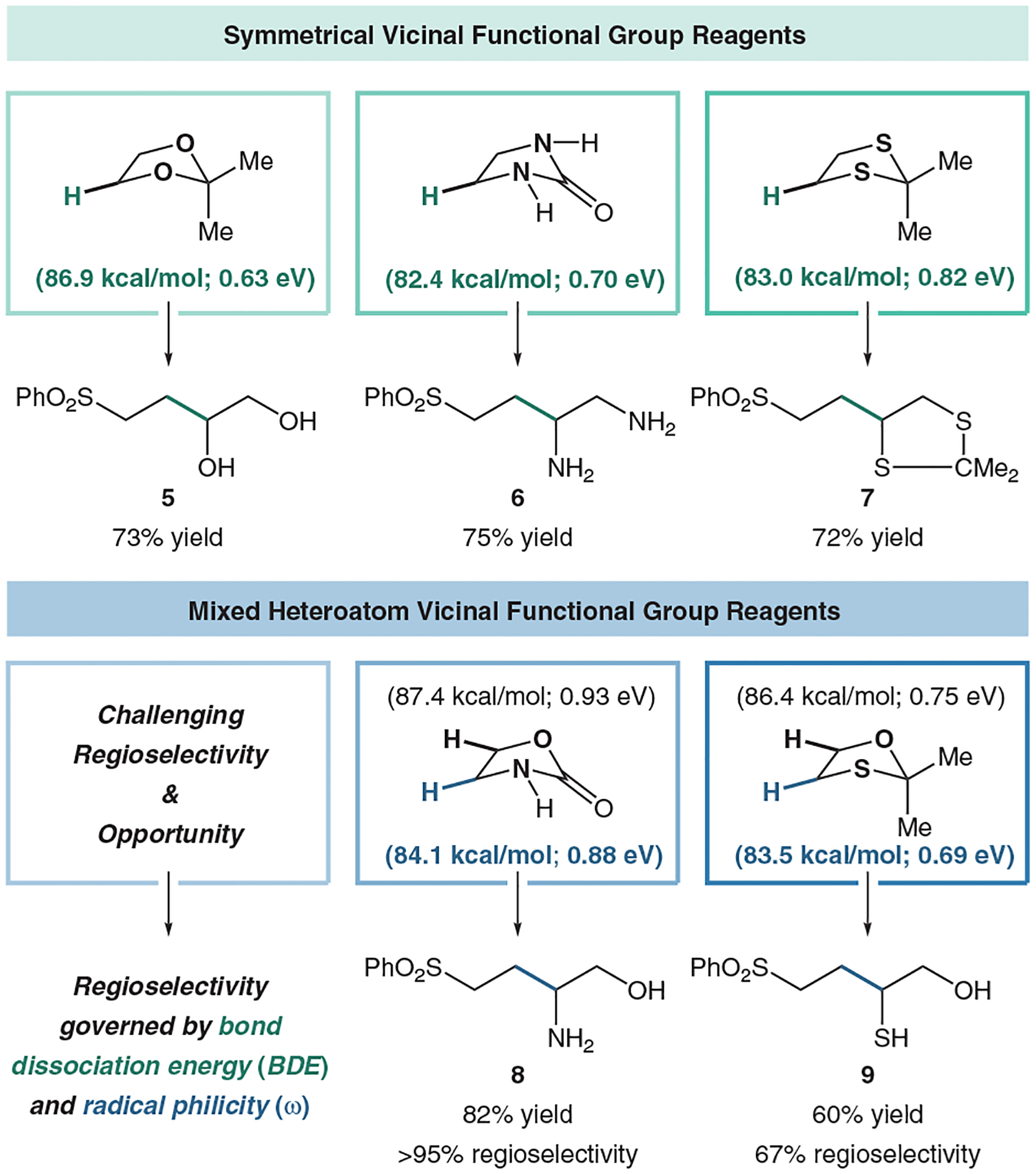
Other reagents to access vicinal functionality

Furthermore, we thought we could expand this logic to access not only 1,2-diols, but also 1,2-diamines, 1,2-amino alcohols, and 1,2-thiol alcohols (Figure 10). Symmetrical diamine reagent imidazolidinone coupled efficiently with phenyl vinyl sulfone in 75% yield (**6**). Likewise, 2,2-dimethyl-1,3-dithiolane coupled in 72% yield (**7**). However, amino alcohol and thiol alcohol reagents provided a new challenge. Mixed heteroatom reagents provide two unique C–H bonds for abstraction, in turn yielding two regiomeric products. Using the same computational methods as for the symmetrical reagents, we could predict regioselectivity based on BDE. After exploring a variety of amino alcohol reagents, oxazolidinone proved to be the best reagent due to ease of deprotection, cost-effectiveness, and favorable computational calculations. We calculated a 3.3 kcal/mol difference between the α-amide C–H bond (84.1 kcal/mol) and the α-acyloxy C–H bond (87.4 kcal/mol). Experimentally, we observed an 82% yield and 100% regioselectivity towards the lower C–H bond (**8**). Using the same logic, we used 2,2-dimethyl-1,3-oxathiolane for our thiol alcohol reagents because we calculated a large BDE difference between the α-thiol C–H bond (83.5 kcal/mol) and the α-oxy C–H bond (86.4 kcal/mol). While coupling yield was competent (60% yield), regioselectivity was modest (67% regioselectivity) towards the lower C–H BDE (**9**). We turned to radical philicity calculations to potentially explain these trends. We found that the α-thiol radical was slightly more nucleophilic than the α-oxy radical, with electrophilicity values of 0.69 eV and 0.75 eV, respectively. A similar discrepancy is seen regarding the amino alcohol reagent (0.88 eV vs 0.93 eV, respectively). This indicates to us that the regioselectivity in the thiol alcohol case is not governed solely by C–H BDE or radical philicity, launching a new opportunity for our lab to explore other computationally and experimentally driven approaches to solve this interesting selectivity challenge.

**Figure 11:**
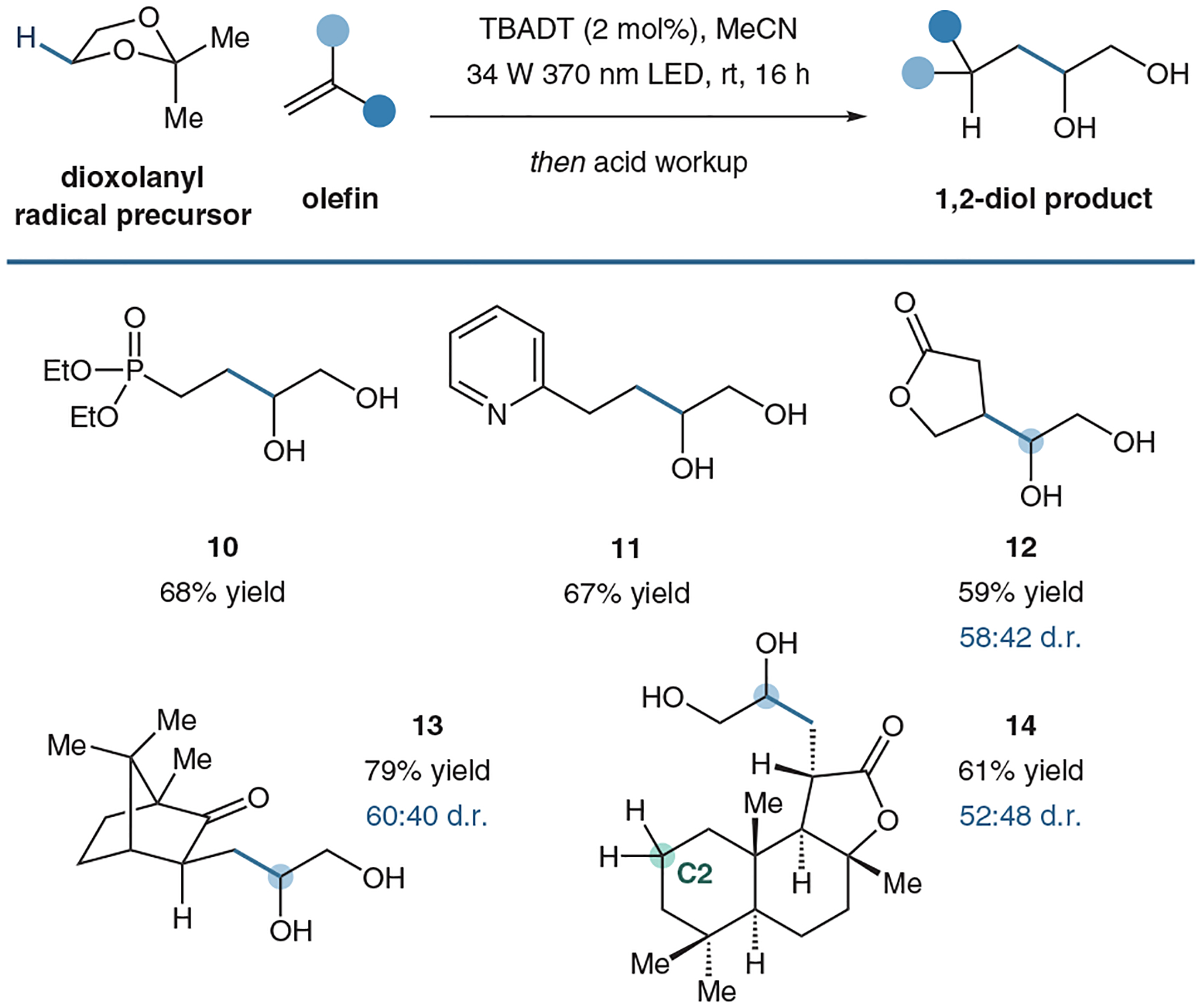
Selected substrate scope

Once our optimized designer-masked diol reagents were in hand, the reaction was implemented on a variety of different electron-deficient olefins including vinyl phosophonates, vinyl amides, vinyl pyridines, and α,β-unsaturated ketones and esters (**10**–**13**, Figure 11). Excitingly, we can tolerate a coupling with substrates that also have labile C–H bonds. For example, lactones are known to readily engage in α-oxy HAT abstraction. Additionally, D-sclareolide is well-known to be susceptible to HAT processes at the C2 position using TBADT as a catalyst (**14**).^54^

However, in both cases, we observe high coupling without any observation of cross selectivity. This highlights the chemoselectivity of our designer acetal reagent. The low BDE of our acetal reagent outcompetes other weak C–H bonds that are ubiquitous in organic molecules (90–105 kcal mol^–1^).^53^

**Figure 12:**
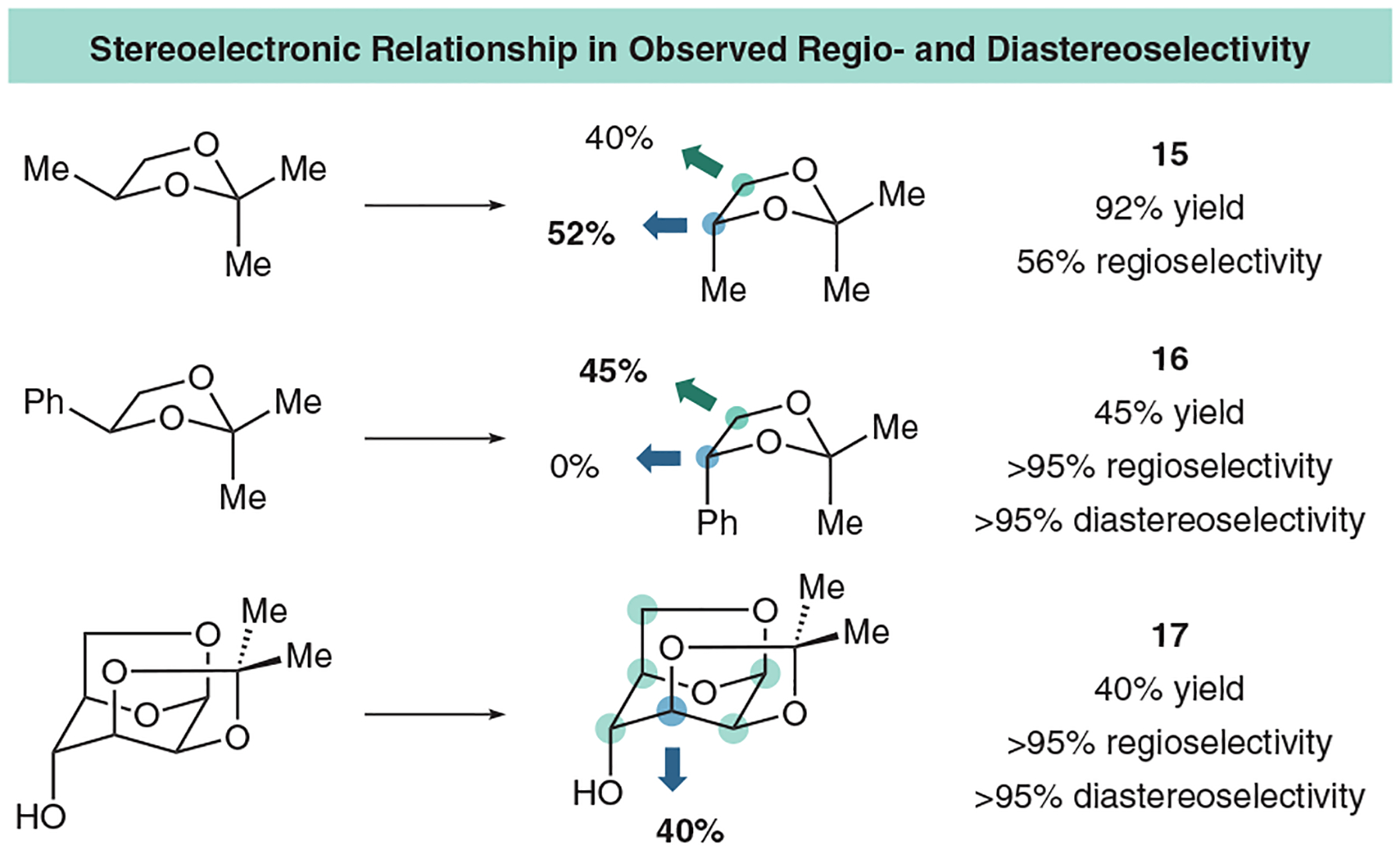
Selected substituted acetal scope

We can also activate pre-oxidized diol motifs and synthesize complex diols in a C–C bond-forming reaction (Figure 12). Knowing that radical reactions can be sensitive to steric bulk, we were curious about the competition between steric bulk and BDE. For example, methyl-substituted acetal had slight regioselectivity towards the more substituted methine carbon (BDE 87.1 vs 85.8 kcal mol^–1^; **15**). However, more sterically encumbered phenyl-substituted acetal had complete regioselectivity towards the less sterically hindered methylene carbon despite large differences in BDE (87.2 vs 75.0 kcal mol^–1^; **16**). Excitingly, we could couple with complex sugar substrates as well with multiple hydridic C–H bonds in a regioselective fashion. Additionally, because the dioxolane here is bound within a bicyclical structure, radical addition occurs at only one face, giving the diastereoselective product **17**. This is in line with work done by Taylor and coworkers, wherein they use borinic acids to activate sugar motifs for HAT processes.^55^

**Figure 13:**
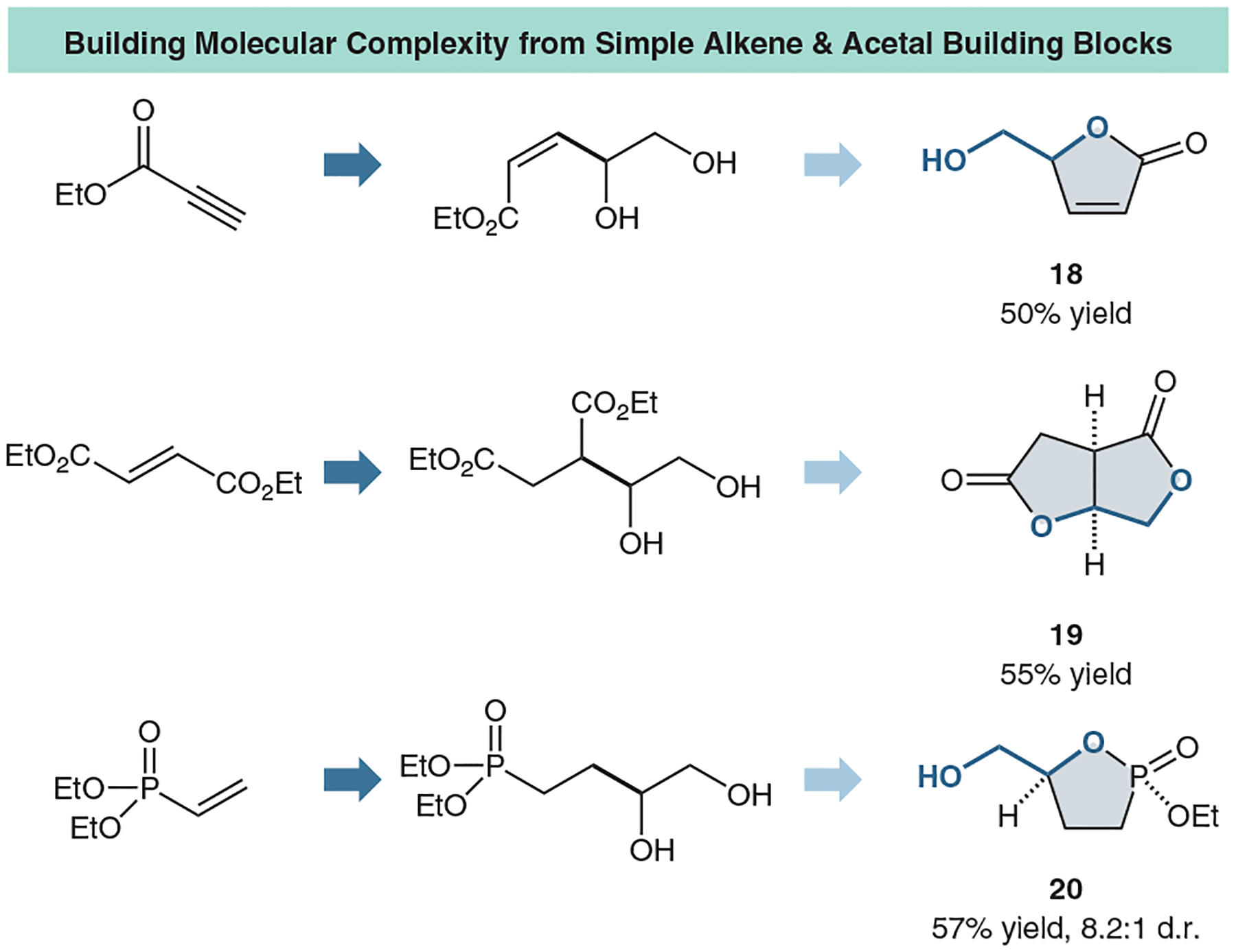
Selected lactonization scope

Lastly, we demonstrated the value of our coupling by engaging it in an *in situ* cyclization by coupling with arylates, propiolates, and ethylene malonates (Figure 13; **18** and **19**). In a one-step procedure, we can access a wide variety of unique scaffolds. In all cases, the five-membered lactone is synthesized rather than the six-membered, following the expected reaction kinetics for ring-closing reactions. Excitingly, this is not only limited to the synthesis of lactones. Chiral cyclic phosphonates can also be synthesized very rapidly in two steps (**20**).

We have demonstrated a unified approach to vicinally functionalized motifs like 1,2-diols, 1,2-amino alcohols, 1,2-diamines, and 1,2-thiol alcohols through the use of acetal-like reagents. We designed a variety of different reagents using computationally driven BDE and radical polarity calculations.

With the results of this initial study in hand, we are excited to further enhance the value of these acetal-like reagents by taking advantage of important asymmetric C–C bond-forming frameworks. Furthermore, oxidation of alkenes to provide dihalogenated motifs is a growing area of interest due to favorable pharmaceutical properties and their use as a versatile synthetic intermediate. A complementary C–C bond-forming approach to build vicinally functionalized dihalogen motifs via an α-halogen C–H process would be extremely valuable to the broader synthetic community.

## 4 Conclusion and Outlook

In summary, this perspective highlights our recent developments in both generating dioxolanyl radicals and implementing them in the syntheses of highly oxidized motifs such as ketones and alcohols. Dioxolanyl radicals can be thought of as dual-use reagents, as they are able to prevent decomposition like decarbonylation, as well as enabling otherwise difficult chemical reactivities. Our fluorinated dioxolane-based carboxylic acid enabled coupling towards electrophilic targets that are traditionally challenging to access using previously established methods. In contrast, selective radical generation on the dioxolane backbone gives opportunities to access 1,2-diol motifs in a C–C bond-forming approach. We can expand this system to other saturated heterocycles to access other vicinally functionalized motifs like diamines, amino alcohols, and thiol alcohols. Nearly a century after their introduction as a protecting group, dioxolanes have been realized as a powerful, synthetic building block that can be harnessed to install highly oxidized motifs for the complex synthesis of drugs and natural products.
